# Transition from glass to digital slide microscopy in the teaching of oral pathology in a Brazilian dental school

**DOI:** 10.4317/medoral.19863

**Published:** 2014-08-17

**Authors:** Felipe-Paiva Fonseca, Alan-Roger Santos-Silva, Márcio-Ajudarte Lopes, Oslei-Paes de Almeida, Pablo- Agustin Vargas

**Affiliations:** 1 DDS, MSc, Oral Diagnosis Department (Pathology and Semiology) – Piracicaba Dental School, State University of Campinas – UNICAMP, Brazil; 2 DDS, PhD, Oral Diagnosis Department (Pathology and Semiology) – Piracicaba Dental School, State University of Campinas – UNICAMP, Brazil; 3 DDS, PhD, FRCPath, Oral Diagnosis Department (Pathology and Semiology) – Piracicaba Dental School, State University of Campinas – UNICAMP, Brazil

## Abstract

Objectives: Several medical and dental schools have described their experience in the transition from conventional to digital microscopy in the teaching of general pathology and histology disciplines; however, this transitional process has scarcely been reported in the teaching of oral pathology. Therefore, the objective of the current study is to report the transition from conventional glass slide to virtual microscopy in oral pathology teaching, a unique experience in Latin America. Study Design: An Aperio ScanScope® scanner was used to digitalize histological slides used in practical lectures of oral pathology. The challenges and benefits observed by the group of Professors from the Piracicaba Dental School (Brazil) are described and a questionnaire to evaluate the students’ compliance to this new methodology was applied. Results: An improvement in the classes was described by the Professors who mainly dealt with questions related to pathological changes instead of technical problems; also, a higher interaction with the students was described. The simplicity of the software used and the high quality of the virtual slides, requiring a smaller time to identify microscopic structures, were considered important for a better teaching process. Conclusions: Virtual microscopy used to teach oral pathology represents a useful educational methodology, with an excellent compliance of the dental students.

** Key words:**Digital microscopy, virtual microscopy, dental education, virtual slides, oral pathology.

## Introduction

In the last decades there has been a huge technologic development used in practically all human activities, including education. Many dental and medical specialties have benefited from the use of these new tools in the teaching process, but the microscopic study of human tissues and diseases has remained an exception. Since the improvement in the manufacture of lenses in the beginning of the 19th century, microscopes have been used for the study of pathology, with no significant development thereafter (1-3).

However, in the last decade of the 20th century, the concept of virtual slides that perfectly emulate a traditional microscope and glass slides emerged, making it possible to examine histological sections at different magniﬁcations without any loss of quality. This new technology brought high expectations to the field of pathology, not only in terms of diagnosis but also facilitating multicenter conferences, international discussions, improving research analyses and shedding new light on academic education methodologies (1,2,4-6). Hence, many dental and medical schools worldwide have looked forward to implementing this new technology for teaching pathology and histology in their regular undergraduate courses. The experiences already obtained show a significant improvement in the students’ devotion and enthusiasm to study pathology, indicating that the use of virtual slides now represents a very important tool to be used in pathology teaching (7-12).

The Piracicaba Dental School of the State University of Campinas is one of the most traditional Brazilian groups of oral pathology and oral medicine, with over fifty years of activity including academic education, clinical/histological diagnosis and the treatment of oral diseases and research. This group is pioneering in the acquisition and use of digital slide scanners in Brazilian Dental Schools, and the first to introduce this technology into its regular academic teaching of oral pathology. Therefore, the aim of the current manuscript is to share the experience of our group with the transition from conventional glass slide microscopy to digital pathology, emphasizing the difficulties and benefits experienced by the students, lecturers and institution.

## Material and Methods

The current study was approved by the ethical committee of our institution (Protocol number CEP/FOP 002/2014).

Oral pathology for undergraduate students at the Piracicaba Dental School consists of a one-year course given during the second year of dentistry; this is provided for 80 students yearly. The course comprises 21 practical classes of histopathology distributed throughout the year, including both general and oral pathology. These activities have traditionally been developed in a laboratory containing 40 bifocal microscopes individually used by the students divided in two groups of 40 students each.

**Figure 1:**
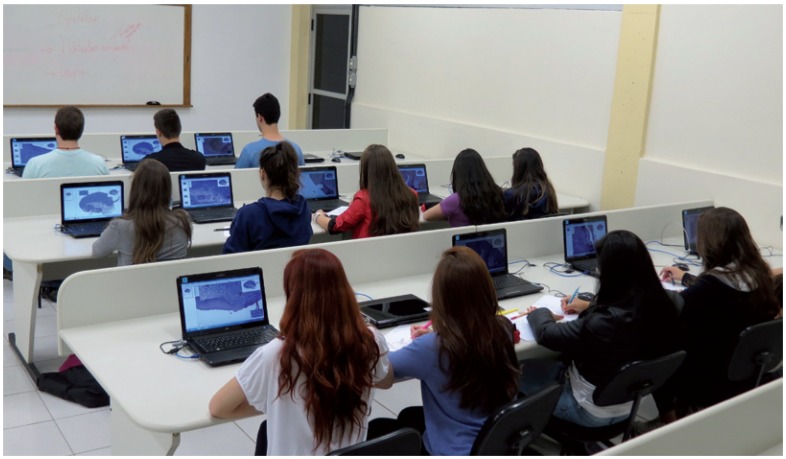
Laboratory of digital pathology containing 40 laptops used individually and independently by each student to study the virtual histological sections.

Following the receipt of the Aperio ScanScope CS® Slide Scanner in June 2011, all conventional glass slides used for classes were digitalized and used in a digital pathology laboratory (DPL). The DPL is equipped with 40 desktops where all 100 histological sections, organized in individual folders, which are regularly used in the course, can be assessed by the students during the classes (Fig. 1). The digital slides were scanned at 20x magnification, and automatically stored on one of the Dental School servers. With the support of the computer technology department of our School, a virtual network linking the scanner computer, the institution server, the DLP and the teachers’ office computers was created. In addition to the virtual histological slides, each folder contained macroscopic, clinical and radiographic figures, so that the students could correlate all diagnostic aspects of each case.

**Figure 2:**
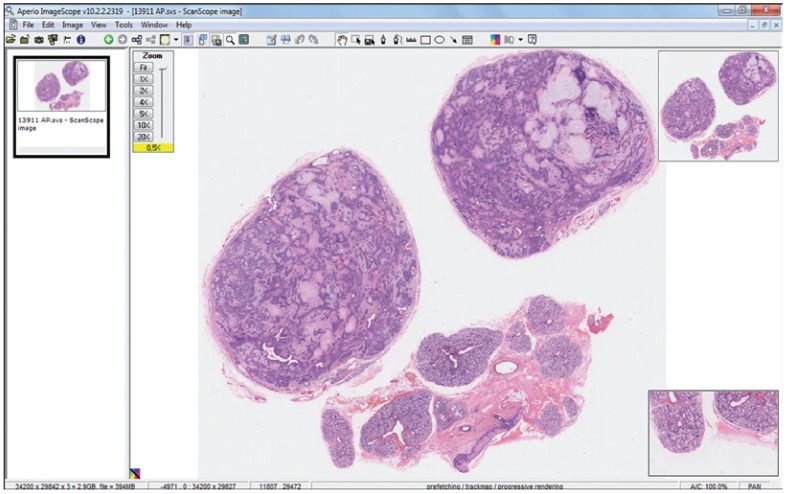
Virtual sections offer a high quality image that can be moved in the x-y axes and can increase magnification up to 40x.

Each student was able to visualize the digital slides independently using the Aperio ImageScope software, moving the images in the x-y axes and increasing magnification using a zoom tool up to 40x (Fig. 2). Two instructors (post-graduate students) were present during the practical teaching activities to provide more support to the students; also, the professors could use a projector to demonstrate the virtual slides to all students when appropriate.

A questionnaire was applied at the end of the course to evaluate the compliance of the students to this new teaching methodology. All participants who voluntarily enrolled in the survey had been taught histology using conventional microscopes and pathology by virtual microscopy. The main objective of the questionnaire was to compare the efficacy of classes for the study of histological material using conventional light microscopes and digital slides. It included questions related to the practicality of using the software, quality of images, need for instructor support, necessity of light microscope teaching, advantages and disadvantages of both methods and motivation. An open-ended comment section was also included so that topics not covered in the questionnaire could be considered.

## Results

Classes using virtual microscopy in the teaching of oral pathology facilitated and improved the activities of the professors involved, as all of their time was used for the teaching process itself, rather than spending time distributing slides or helping students to manage their microscopes. As a consequence, by using the same number of sections, more time became available and macroscopic, clinical and radiographic images could also be included in the session. The use of the same slides by all students also improved the classes, since all students could now identify a given structure, which was not necessarily present in all of the glass slides used traditionally.

**Figure 3:**
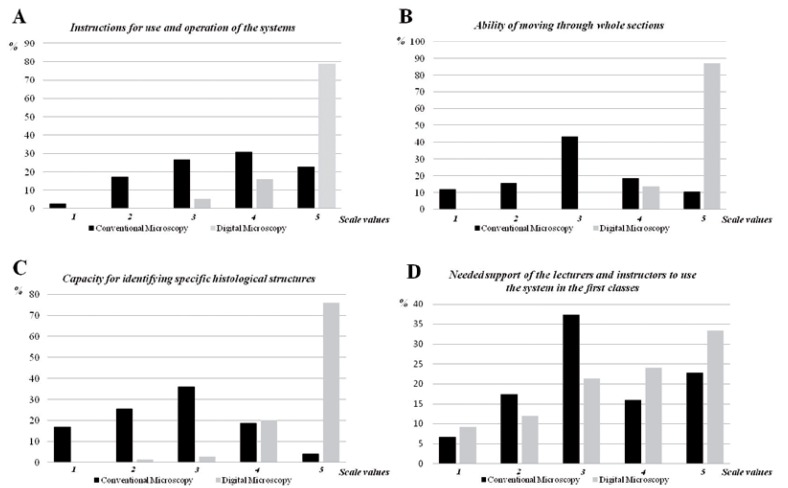
Ratings of student perceptions of conventional and virtual microscopies based on a scale of 1 to 5, where 1 represents the worst performance and 5 the best. Virtual microscopy revealed much better results than conventional glass slide microscopy according to the students A) when the instructions for use and operation of the systems was compared, B) when the ability of moving through whole sections was compared, and C) when the capacity for identifying specific histological structures was compared. D) On the other hand, the questionnaire showed that students needed some support of the lecturers and instructors to use the digital system in the first classes.

Of the 80 students concluding the oral pathology course, 75 answered the questionnaire. Most of them were very enthusiastic with regard to the new teaching methodology, and stated that it increased their interest in the classes and consequently improved their learning. According to 98.7% of the students, the new digital system was much easier to manipulate, thus requiring less time to identify histological structures, and also provided better definition when compared to conventional glass slides ( Table 1). However, the students reported that they needed the help of the professors and instructors to deal with the software and identifying the structures at the first time of use (Fig. 3). It is interesting to consider that, although the results of all questions favored digital slides, 56% of the students suggested that conventional microscopy should not be completely eliminated and stated that brief contact with traditional glass slides and regular microscopes was still important ( Table 2).

**Table 1:**
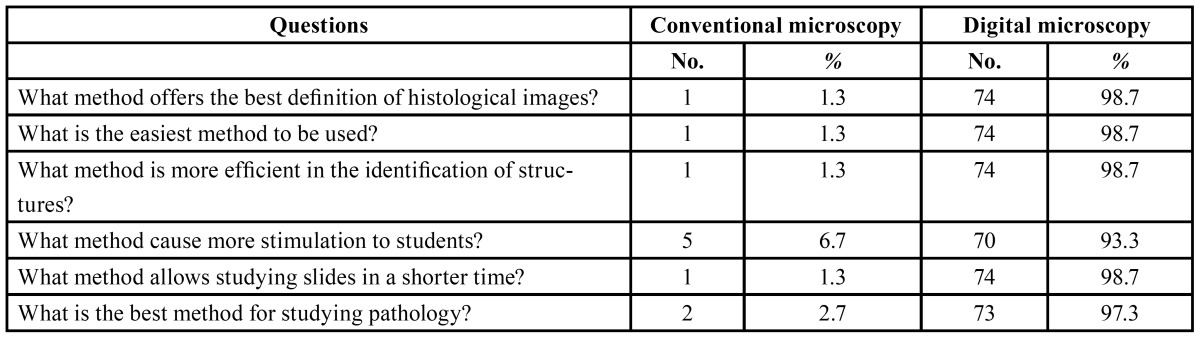
Opinion of the students about what would be the best methodological approach.

**Table 2:**
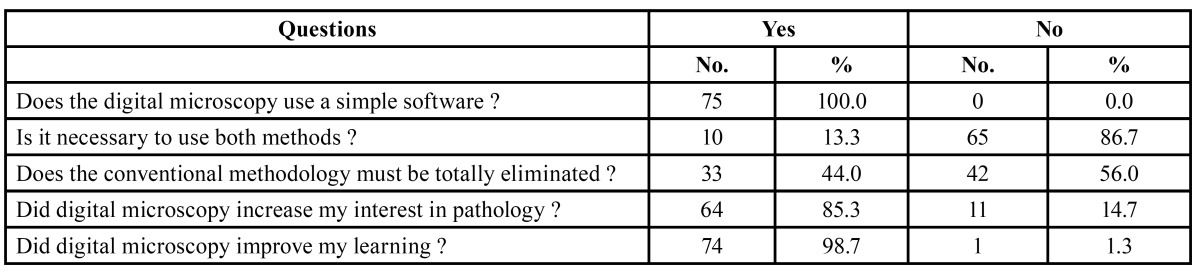
Opinion of the students about some of the digital microscopy features.

The open-ended comment section was rarely used by the students, and they most commonly suggested small improvements, such as the inclusion of a mouse for the laptops and the substitution of old sections by new ones. All suggestions were considered relevant, simple to implement and have already been done by the school and the department. Finally, all of the teachers involved to date, are enthusiastic regarding the new methodology, considering this an important step for the teaching of pathology.

## Discussion

Although several medical and dental schools have described their experience in the transition from conventional to digital microscopy in the teaching of general pathology and histology disciplines, this transitional process has scarcely been reported in the teaching of oral pathology; however, similar to that observed in the current survey, all previous descriptions found a significant compliance of dental students with this new methodology (5,9,10). Moreover, despite Rosas *et al*. (13) described the use of computed facilities in the teaching of histology, where they used static pictures for visualizing histological structures, to the best of our knowledge, the current manuscript describes the first experience of a Latin American Dental School group teaching oral pathology with whole virtual slide technology.

In contrast to Weaker *et al*. (11) who aimed to incorporate digital slides to supplement and not replace conventional light microscopy, we decided after 3 years of experience, to completely abandon the use of glass slides for undergraduate dental students, similar to other groups (5,9). During this transitional period, our students experienced both methodologies, and were able to more precisely compare the advantages and disadvantages of them. If students, out of curiosity or for any particular reason, want to learn how to use a conventional microscope, we still offer them this opportunity in our research laboratories in extra lessons.

As previously described by others in both general and oral pathology teaching (7-10), the signiﬁcantly higher ratings given to the efficiency of the virtual microscopy in our questionnaire clearly demonstrate the enthusiastic endorsement of the students of this new methodological approach, which could be explained by the innumerous advantages of virtual slides. The easy management of the software and high quality of the digital image not requiring from the students any further effort to find adequate focus or light adjustment for the visualization of structures, which was described by over 90% of our students and by the great majority of the students from other groups, shows that the virtual slides are preferred by students, especially those with inferior technical competence with classical microscopes (5,6,8). In addition, digital slides overcame the common problem seen with the use of glass slides of the structures that were supposed to be identified during microscopic study not being visible in all sections, which meant that students had to share slides, leading to slide breakage and loss (4,6,11). It is important to emphasize that many biopsies are very small in oral pathology, precluding the preparations of 40 or more slides, whereas for digital slides you only need one section that will be shared by as many students as necessary.

It is not necessary to state that new generations of students have enormous ability and motivation to use digital devices. Hence, the management of digital slides should be simple for them, leading to active and independent learning, and also facilitating direct interaction among themselves for discussion of the case in study (4,6,10). However, the instructor is still necessary to facilitate the identification of structures and to clarify the use of the software, especially in the first classes, which has also been reported in the literature (7). On the other hand, as also emphasized by different groups (5,6), the professors thought that their time can be more effectively used because questions from students are now generally concerned with the pathological changes instead of technical problems; also, they can now demonstrate histological structures simultaneously for all students with the help of a screen projector, avoiding the necessity of showing this individually to each student in his/her microscope. In fact, all of this practicality has significantly decreased the time necessary for the study of a particular case, and has permitted the incorporation of additional cases in the same class alongside the use of clinical, macroscopic and radiographic images.

The high expense required for the acquisition of a slide scanner still represents a major limitation for the creation of a digital teaching laboratory. However, this initial expense can be significantly compensated by the following savings in microscope management, glass slides processing and storage and the necessity of an exclusive laboratory space (1,4,6). It is also possible for various departments or institutions to share the same scanner, or to use similar teaching materials.

Various groups have described the use of digital slides together with website facilities, making the virtual sections available for students at any time and at any place where they have internet access (6,9,10). Although our system has currently being working only in the school laboratory, our future objectives include making all sections available in a web database for access at any time by students, overcoming time and space constraints. Furthermore, the update of old sections used in traditional glass slide classes and the development of digital tests represent further future perspectives of our group. However, we would like to have the advantages of these web facilities tested first before full implementation.

In summary, the use of virtual microscopy in the teaching of oral pathology represents a relevant improvement, increasing the motivation of students and the quality of the teaching process. The high definition of the histological images, software simplicity, use of additional materials such as clinical and radiographic images, and the facility of interactions between students and teachers are some positive points that have been emphasized by the students. Moreover, the time of lecturers and students is more effectively used, resulting in a better and more interesting teaching process. As shown by us and reported by other institutions, the advantages of virtual microscopy in teaching of histopathology may lead to the complete replacement of conventional microscopy by virtual slides in the near future.
